# Characterization of a novel thermophilic metagenomic GH5 endoglucanase heterologously expressed in *Escherichia coli* and *Saccharomyces cerevisiae*

**DOI:** 10.1186/s13068-022-02172-4

**Published:** 2022-07-07

**Authors:** Juan-José Escuder-Rodríguez, María González-Suarez, María-Eugenia deCastro, Almudena Saavedra-Bouza, Manuel Becerra, María-Isabel González-Siso

**Affiliations:** Grupo EXPRELA, Centro de Investigacións Científicas Avanzadas (CICA), Departamento de Bioloxía, Facultade de Ciencias, Universidade da Coruña, 15071 A Coruña, Spain

**Keywords:** Endoglucanase, Xylanase, Metagenomics, Yeast, Biofuel, Saccharification

## Abstract

**Background:**

Endoglucanases from thermophilic microorganisms are a valuable resource as they can be used in a wide variety of biotechnological applications including the valorisation of biomass and the production of biofuels. In the present work we analysed the metagenome from the hot spring Muiño da Veiga, located in the northwest of Spain (in the Galicia region), in search for novel thermostable endoglucanases.

**Results:**

Sequence analysis of the metagenome revealed a promising enzyme (Cel776). Predictions on protein structure and conserved amino acid sequences were conducted, as well as expression in heterologous systems with *Escherichia coli* and *Saccharomyces cerevisiae* as the host. Cel776*Ec* was correctly expressed and purified by taking advantage of the His-Tag system, with a yield of 0.346 U/mL in the eluted fraction. Cel776*Sc* was expressed extracellulary and was easily recovered from the supernatant without the need of further purification, requiring only a concentration step by ultrafiltration, with a significantly higher yield of 531.95 U/mL, revealing a much more suitable system for production of large amounts of the enzyme. Their biochemical characterization revealed biotechnologically interesting enzymes. Both Cel776*Ec* and Cel776*Sc* had an optimal temperature of 80 °C and optimal pH of 5. Cel776*Ec* exhibited high thermostability maintaining its activity for 24 h at 60 °C and maintained its activity longer than Cel776*Sc* at increasing incubation temperatures. Moreover, its substrate specificity allowed the degradation of both cellulose and xylan. Whereas Cel776*Ec* was more active in the presence of calcium and magnesium, manganese was found to increase Cel776*Sc* activity. A stronger inhibitory effect was found for Cel776*Ec* than Cel776*Sc* adding detergent SDS to the reaction mix, whereas EDTA only significantly affected Cel776*Sc* activity.

**Conclusions:**

Our study reports the discovery of a new promising biocatalyst for its application in processes, such as the production of biofuel and the saccharification of plant biomass, due to its bifunctional enzymatic activity as an endoglucanase and as a xylanase, as well as highlights the advantages of a yeast expression system over bacteria.

**Supplementary Information:**

The online version contains supplementary material available at 10.1186/s13068-022-02172-4.

## Background

Cellulose is the most abundant polymer on Earth and the main component of plant biomass. It normally presents itself in fibres associated to other biopolymers, namely, hemicelluloses and lignin, in a complex structural matrix in plant cell walls [1]. This complex matrix limits the extent and rate of utilization of plant biomass, usually requiring harsh pre-treatments and the action of multiple enzymes to perform the full breakdown of the structure [2]. Although multiple enzymatic activities are responsible of the conversion of cellulose into simpler molecules, collectively known as cellulases, β-1-4-endoglucanases (EC 3.2.1.4) are especially important as they act on the cellulose chain cleaving internal glycosidic bonds and releasing oligosaccharides of different lengths. These are further hydrolysed by other cellulolytic enzymes, such as non-reducing end cellobiohydrolases (EC 3.2.1.91), reducing-end cellobiohydrolases (EC 3.2.1.176) and cellodextrinases (EC 3.2.1.74), and finally converted in glucose by β-glucosidases (EC 3.2.1.21) reducing the product inhibition of all the enzymes mentioned before [3]. Endoglucanases have been classified along with other enzymes based on sequence similarity in the Carbohydrate Active Enzymes (CAZy) Database [4] (http://www.cazy.org/) in 12 Glycosyl Hydrolase families: GH5, GH6, GH7, GH8, GH9, GH12, GH44, GH45, GH48, GH51, GH74, and GH124. Endoglucanases have multiple biotechnological applications, and are especially important in the valorisation of agro-industrial by-products [2] and in the production of biofuels combined with β-glucosidases to produce glucose later fermented into (bio)ethanol [5], as these two applications are directly targeting environmental challenges. In the textile industry there are several enzymatic processes, such as biostoning (to give a wash-down look on cotton clothes) and biopolishing (softening and brightening of cotton surfaces) that remove cellulose fibres and replace more harsh treatments [6]. Similarly, detergent formulations can include endoglucanases, also brightening and softening cotton fabrics [7]. They are also employed in the food and brewing industries, improving digestibility of food and decreasing viscosity, and increasing fermentable compounds for the elaboration of alcoholic drinks [6]. These properties have also been exploited in the animal feed industry, enhancing digestibility and nutrient bioavailability [6]. The pulp and paper industry has many uses for endoglucanases including biopulping, treatment of pulp wastes, deinking and removal of pollutants from paper [6]. Other reported uses include waste management, improvement of soils for agriculture and extraction of bioactive compounds, pigments and oils from plants [3]. Many of these applications are benefited from the use of combinations of various enzymes (enzyme cocktails) or multifunctional enzymes, as the plant biomass is composed of a complex matrix of cellulose, hemicelluloses and lignin [8]. Industrial processes such as biofuel production, food processing, treatments in the pulp and paper industry and production of nutraceuticals have been explored in this context, among others [9, 10]. In this biotechnological context, cellulases from thermophilic microorganisms have added advantages over their mesophilic counterparts. First of all, they are able to withstand the harsh conditions associated with industrial processes. This in turn reduces costs related to the need of cooling large amounts of water or other solvents. Moreover, the diffusion rates and solubility of reagents are higher at high temperatures and the risks of contamination are reduced [11]. Sources for these thermophilic enzymes are varied and included habitats, such as terrestrial hot springs, hydrothermal vents, compost and hydrocarbon reservoirs, among others [12]. Of these, terrestrial hot springs are one of the most common sources of thermophilic enzymes ([13], since the pioneering work describing the first thermophilic organisms in Yellowstone National Park (USA) [14] and the isolation of the *Thermus aquaticus* polymerase that allowed the development of the PCR technique. As with many extremophilic microorganisms, thermophiles are difficult to grow in laboratory conditions and culture independent methods such as metagenomics are needed for assessing their metabolic potential [15]. Lists of thermophilic cellulases found by metagenomics [12] and characterized thermophilic cellulases [3] are available, and novel cellulases found following this strategy continue to be discovered [16, 17] showing the interest for these biocatalysts. Moreover, the discovery of multifunctional enzymes that can act on more than one biomass polymer [18–20], and the characterization of microbial consortiums that produce multiple lignocellulolytic enzymes [21] are also recent research focuses. The development of high-throughput Next Generation Sequencing (NGS) technologies, and more specifically of shotgun metagenomics, has allowed to directly sequence the large number of genomes present in environmental samples, using multiple templates in parallel without targeting specific genes [15, 22]. The method relies on annotated data (reference genomes and gene and protein databases), as gene search and functional annotation using bioinformatic tools is based on alignment and homology to deposited sequences [15, 22, 23, 24]. Nevertheless, sequence based metagenomic studies also face various challenges, including quality and length of the reads generated, and amplification bias and other artifacts, such as chimeric sequences and secondary structures [22]. It is also important to consider that the success of the method heavily relies on the quality of the database annotation and is limited to find somewhat similar sequences in known protein families [23]. When the objective is to bioprospect for novel gene products, the short reads can be linked together into a bigger sequence (contig). Due to the high computational demand of methods based on overlapping reads, many de novo assemblers use instead a de Bruijn graph approach [24]. Predicted Open Reading Frames (ORF) within the reconstructed contigs can then be submitted and aligned to known sequences deposited in annotated databases, allowing the identification of ecological or biotechnological functions of interest. Finally, is important to remark that functional characterization of predicted gene products is still necessary to confirm the results of the in silico analysis [12]. In this regard, the selection of the heterologous expression system has become increasingly important in the context of biotechnological driven bioprospections, as factors such as thermostability, purification from intracellular or extracellular medium and enzyme kinetics are all affected by it [25]. Differences such as the ability to perform post-translational modifications including glycosylation, and high levels of enzymatic yield make hosts such as the yeast *S. cerevisiae* attractive for the expression of recombinant thermophilic enzymes [1–5, 7–10, 12, 16–21,26].

In the present work, we conducted a metagenomic sequence-based screening for genes that were predicted to encode putative β-1-4-endoglucanases with the premise that the metagenome from a hot spring was expected to include thermophilic variants of these biocatalysts. Cloning and expression in two different heterologous hosts was performed, and purification and biochemical characterization followed, to confirm the in silico analysis with functional assays.

## Results

### Bioinformatic pipeline

From the original 170,738,281 reads with a length of 100 bp from the Illumina HiSeq sequencing used for assembly, our pipeline (IDBA-UD assembly into contigs, Deconseq decontamination of NCBI GRCh38 human genome database and MG-RAST upload) resulted in 365,318 contigs with an average length of 780 bp. The search for endoglucanases in the contig sequences using the MG-RAST server and the Subsystems database resulted in 19 contigs containing putative endoglucanases. Of those, three contigs contained predicted complete ORF (with an initial methionine and a STOP codon). These were named based on putative activity and numbered after the contig they were found in as follows: Cel232; Cel652 and Cel776. Through BLASTp alignment to the NCBI NR database, it was found that Cel232 had a 99% identity with an endoglucanase from *Dictyoglomus thermophilum* and Cel652 had a 97% identity with an endoglucanase from *Fervidobacterium nodosum*, whereas Cel776 had a 73% identity with a glycoside hydrolase family 1 protein from *Fervidobacterium pennivorans*. Based on the lower identity of Cel776 to known proteins, it was selected for further characterization.

### Cel776 sequence analysis

**Fig. 1:**
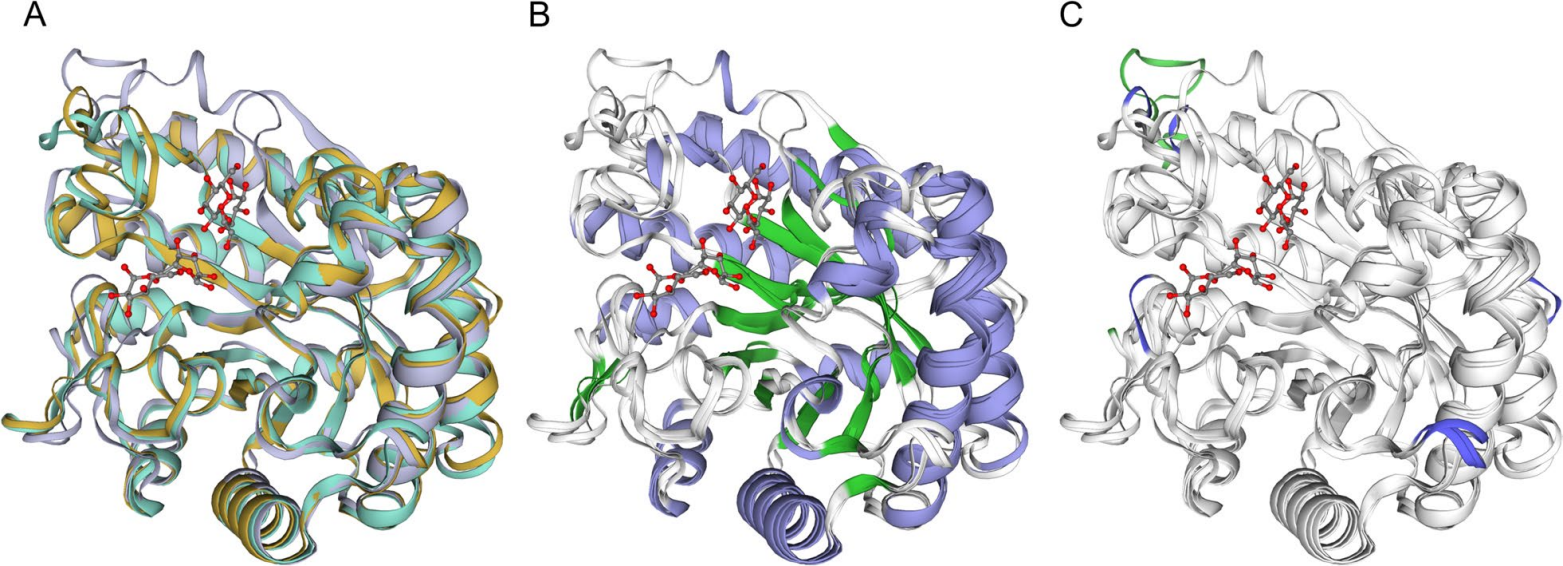
Structural protein alignment of three models for Cel776 generated with SWISS-MODEL. **A** Model 1 was constructed using as template 3rjy.1.A, the endoglucanase from *Fervidobacterium nodosum* (*Fn*Cel5A) in complex with substrate alpha-d-glucopyranose (green). Model 2 was constructed using as template 3azr.1.A, an inactive mutant of the endoglucanase from *Thermotoga maritima* [*Tm*Cel5A (E253A)] in complex with cellobiose (yellow). Model 3 was constructed using as template 4u5i.1.A, an inactive mutant endoglucanase from *Clostridium thermocellum* [*Ct*Cel5E (E314A)] in complex with xylobiose (light blue). **B** Structural alignment of the three generated models highlighting the secondary structure (alpha helices in blue, beta sheets in green). **C** Structural alignment of the three generated models highlighting the indels. Substrates are represented in ball-and-stick model

**Fig. 2:**
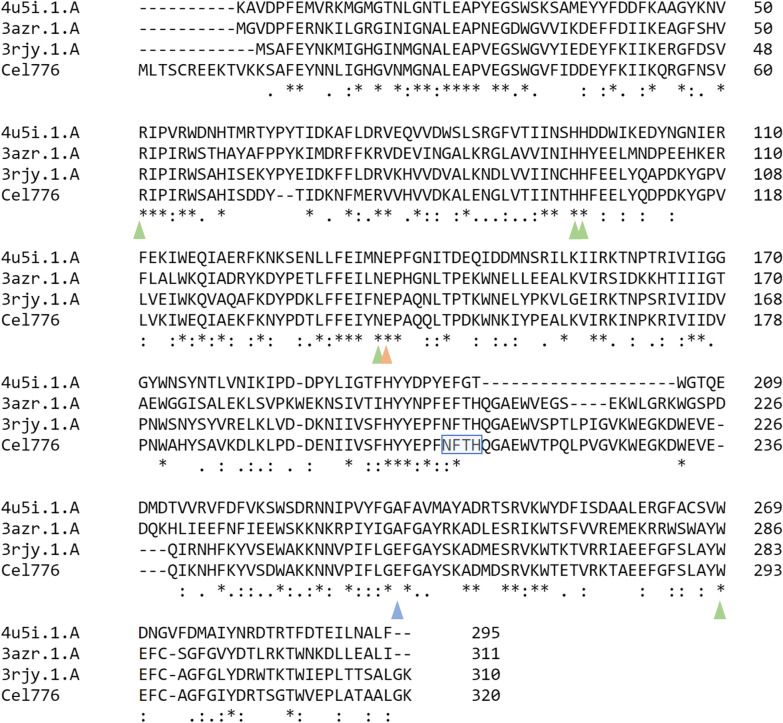
Alignment of Cel776 with GH5 family proteins of known structure. Alignment of Cel776 to sequences in the CAZy database annotated as GH5 with known structure. Green triangles indicate conserved residues of GH5 that have been linked to the catalytic activity of the enzyme. The orange triangle highlights the conserved catalytic residue that act as nucleophile. The blue triangle indicates the conserved catalytic residue that acts as proton donor (mutated in two of the proteins that are rendered inactive). The blue box indicates a predicted *N*-glycosylation site in the Cel776 sequence. 4u5i.1.A: sequence of inactive mutant *Ct*Cel5E (E314A) bifunctional cellulase/xylanase from *Clostridium thermocellum*. 3azr.1.A: sequence of inactive mutant *Tm*Cel5A (E253A) multifunctional endoglucanase from *Thermotoga maritima*. 3rjy.1.A: sequence of *Fn*Cel5A endoglucanase from *Fervidobacterium nodosum* Rt17-B1

Cel776 gene product was predicted to be 320 amino acids long, with a theorical molecular weight of 37,232.31 Da and an isoelectric point of 5.67. BLASTp against the NR protein database resulted in the best hit for a glycoside hydrolase family 5 protein from *Fervidobacterium* sp. 2310opik-2 (ID: WP_164541660.1) with a score of 531 bits and 76.69% identity. SWISS-MODEL modelling had the best hit with the template for the crystal structure of FnCel5A from *F. nodosum* Rt17-B1 with a sequence identity of 79.68% and a GMQE of 0.92, whereas the QmeanDisCo Global score was 0.90 ± 0.05. The template is annotated in the CAZy database as a GH5 family protein belonging to subfamily 25. Protein models of Cel776 are given in Fig. 1 generated with SWISS-MODEL. No signal peptide was predicted for the amino acid sequence of Cel776. Key conserved residues in other GH5 family proteins were identified in Cel776 sequence by sequence alignment using Clustal Omega (Fig. 2), including the catalytic pair of Glu144 and Glu260. In the GH5 endoglucanase from *Thermotoga maritima* MSB8 hydrogen bonds between an Arg and His residues with these two catalytic Glu residues are described [27], that in the Cel776 protein would correspond to Arg61 with nucleophile Glu260 and His203 with proton donor Glu144. The conserved Asn143 in Cel776 also forms a stabilizing hydrogen bond with conserved Arg61 and stabilizes the transition state with a hydrogen bond with glucan substrates in the enzyme from *T. maritima*. Trp293 is described to allow glucose-binding in a hydrophobic context in the -1 subsite [27]. These four conserved residues could be identified in the multiple sequence alignment (Fig. 2) and their spatial distribution in the active site is similar across the members of the GH5_25 family [28]. Studies on protein structure bound to the substrates xylobiose and cellobiose and site directed mutagenesis had been conducted on the endoglucanase/xylanase from *C. thermocellum* [28] and with substrates cellotetraose, cellobiose and mannotriose on the GH5 endoglucanase from *T. maritima* [29]. Identified conserved residues important to catalytic activity in these two enzymes where also present in Cel776, including: Asn30, His103, His104, Asn143, Glu144, Tyr205, His212, Trp227 and Trp293. These are thought to be involved in substrate recognition mechanisms in enzymes of GH5 family. Moreover, ScanProsite analysis revealed that aminoacids in position 209 to 212 (NFTH) constitute a predicted N-glycosylation site. A close-up of the active site is provided in Additional file 2: Fig. S1 Haga clic o pulse aquí para escribir texto.

### Cloning and purification

For Cel776*Ec*, correct BP and LR recombination reactions were verified by sequencing using the M13 and T7 sequencing primer pairs, respectively. Cel776*Ec* sequence was correctly cloned in the pDNOR221 and pDEST527 vectors in the corresponding recombination step. In addition, for the LR recombination, no growth on LB plates supplemented with chloramphenicol and ampicillin was observed for each of the clones harbouring the pDEST527 vectors with an insert DNA. Cel776*Sc* was successfully cloned in the *Saccharomyces cerevisiae* BJ3505 strain with the YepFLAG-1 vector as revealed by PCR using specific primers and by sequencing.

**Fig. 3:**
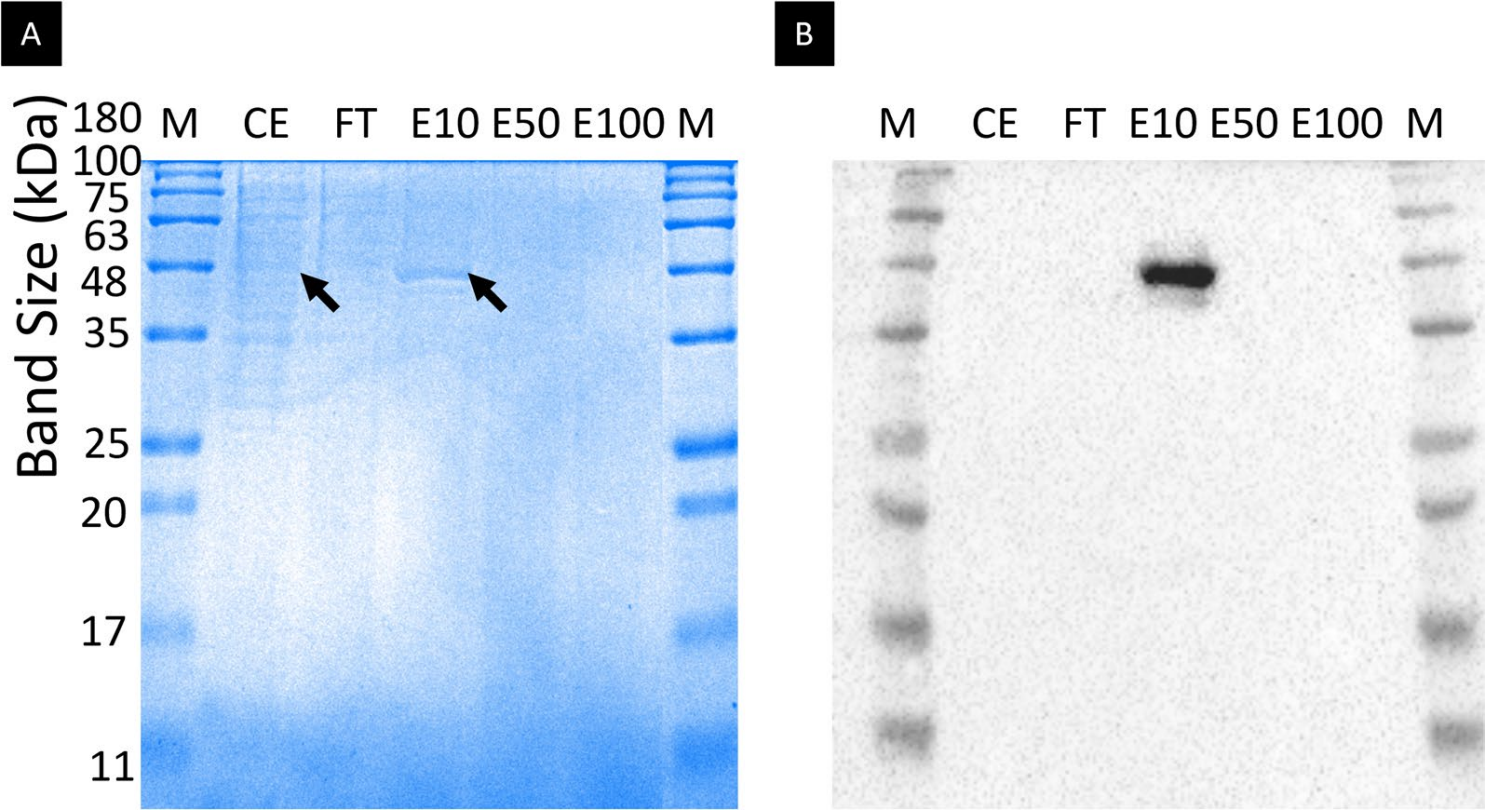
SDS–PAGE and western blot for Cel776. **A** SDS–PAGE of the purification process for the Cel776*Ec* gene product. M: NZYColour protein marker II (NZYTech, Portugal) 2.5 µL; CE: Crude Extract 9 µL; FT: flow-through from the column load and wash steps 9 µL; E10: Elution fraction at 10% elution buffer concentration 18 µL; E50: Elution fraction at 50% elution buffer concentration 18 µL; E100: Elution fraction at 100% elution buffer concentration, 18 µL. Black arrows mark the band corresponding to the Cel776*Ec* protein. **B** Western blot of the purification steps for the Cel776*Ec* gene product. Lanes are named as in **A**

As shown in Fig. 3, the Cel776*Ec* gene product was purified by affinity chromatography taking advantage of the 6xHIS tag in the C-terminus of the protein and then concentrated. The protein was recovered in the elution fraction containing 10% elution buffer, with a yield of 0.346 U/mL.

### Biochemical characterization

**Fig. 4:**
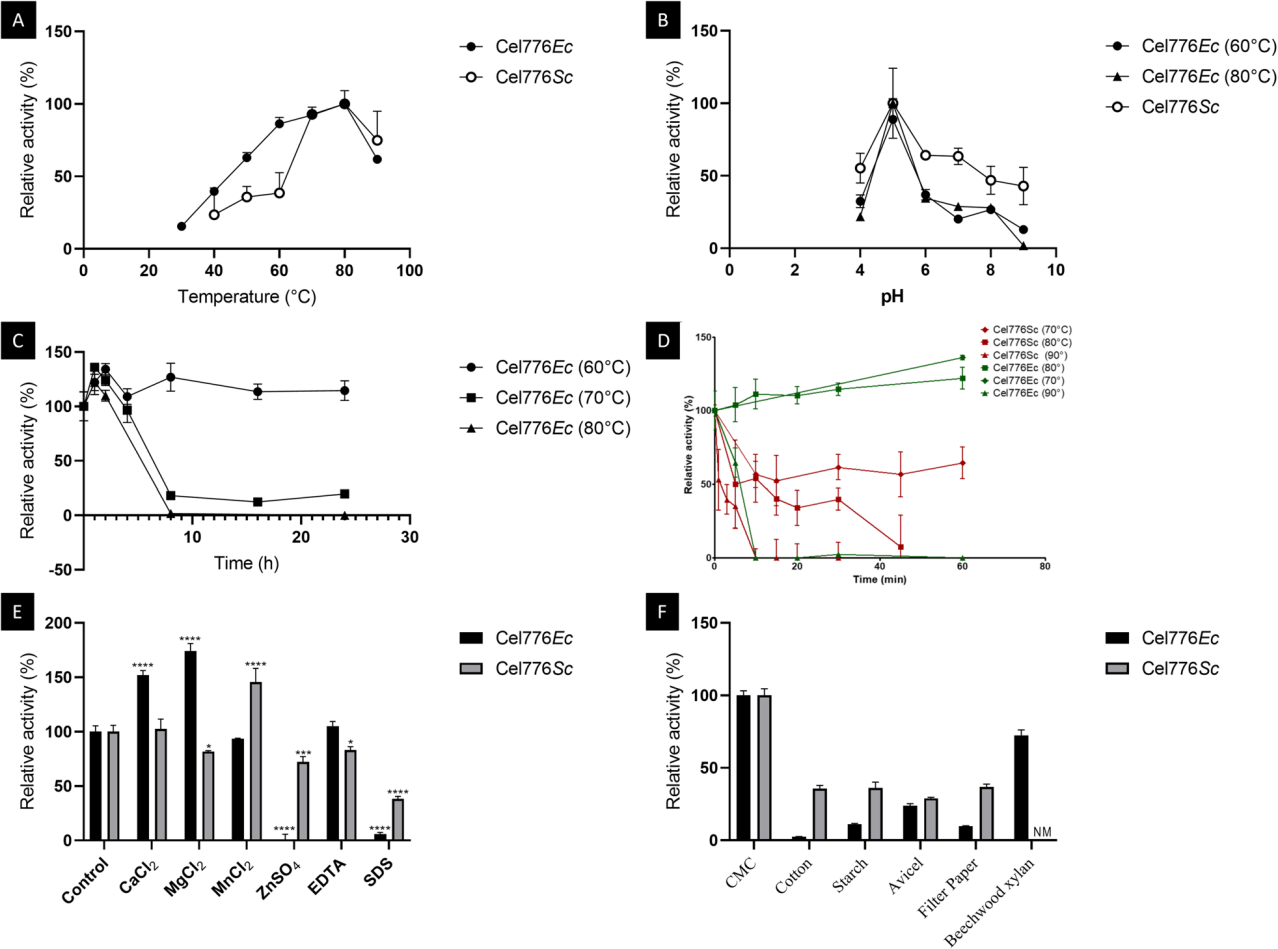
Biochemical characterization of Cel776*Ec* and Cel776*Sc*. All measurements were the result of three independent experiments and an enzyme blank was subtracted to each condition tested. **A** Optimal temperature at pH 5. **B** Optimal pH at temperatures 80 °C and 60 °C for Cel776*Ec* and 60 °C for Cel776*Sc*. **C** Thermal stability at 60 °C, 70 °C and 80 °C for long incubation times (1 h to 24 h) for Cel776*Ec* and **D** thermal stability at 70 °C, 80 °C and 90 °C for short incubation times (1 min to 60 min) for Cel776*Ec* (green) and Cel776*Sc* (red). **E** Effect of additives at 5 mM concentration on the enzymatic activity. An asterisk represents a *p* value lower than 0.05 in a one way ANOVA test for the control and each condition. Two represent a *p* value lower than 0.005; three lower than 0.0005 and four lower than 0.0001. **F** Activity towards alternative substrates. NM: not measured

The optimal temperature for both endoglucanases Cel776*Ec* and Cel776*Sc* was 80 °C (Fig. 4A), with 100% activity representing 0.6917 U/mL. The optimum pH was 5 in both cases. Cel776*Ec* activity was severely reduced with varying pH conditions, and more so at 80 °C than at 60 °C. Cel776*Sc* was also affected by pH variations but to a lesser degree (Fig. 4B), with 100% activity representing 0.5378 U/mL. Cel776*Ec* was found to be thermostable, maintaining its activity for over 24 h at 60 °C and with a half-life of 6.38 h at 70 °C and 3.55 h at 80 °C (Fig. 4C), whereas treatment at 90 °C quickly inactivated the enzyme with a half-life of 6.11 min (Fig. 4D), where 100% activity is 0.9086 U/mL. On the other hand, Cel776*Sc* was less thermostable, as it was found that a 10 min incubation at 90 °C completely inactivated the enzyme, and only 40% activity was retained after incubation at 80 °C for 15 min. Cel776*Sc* maintained its activity around 60% of the non-heated enzyme after incubation for 1 h at 70 °C (Fig. 4D). Some metal ions enhanced Cel776*Ec* enzymatic activity, particularly with the addition of CaCl_2_ and MgCl_2_, and similarly with MnCl_2_ for Cel776*Sc* (Fig. 4E). The non-additive control was stablished as 100% activity (0.6748 U/mL). The addition of EDTA, reduced the activity of Cel776*Sc* but not of Cel776*Ec*. The addition of ZnSO_4_ strongly inhibited the enzymatic activity of Cel776*Ec*, while Cel776*Sc* showed less inhibition by it. Cel776*Ec* was also more susceptible to inhibition by detergents, as SDS addition almost rendered the enzyme inactive, whereas Cel776*Sc* maintained around 40% of its activity (Fig. 4E). Activity towards CMC at optimal temperature and pH was used as a 100% control activity (0.5377 U/mL) compared to alternative substrates. Cel776*Ec* displayed almost no activity towards cotton and very little activity towards filter paper and starch, while Cel776*Sc* did show some activity towards all three substrates. Both enzymes showed activity when the substrate was insoluble microcrystalline cellulose (AVICEL) and Cel776*Ec* was shown to degrade xylan as well (Fig. 4F). Enzyme kinetics for Cel776*Sc* were determined, it followed a classical Michaelis–Menten kinetic with *V*max 0.6686 UE µL^−1^ and *K*m = 0.1716 µg µL^−1^ and compared to other thermophilic GH5 endoglucanases (Table 1). Cel776*Sc* activity in the culture medium was determined as 531.95 U/mL (one enzymatic unit is defined as the amount of enzyme that produces a nanomole of glucose in the assay conditions).

**Table 1:** Comparison of enzymatic parameters of thermophilic GH5 family endoglucanases with Cel776*Ec* and Cel776*Sc*

Enzyme	Source	Heterologous host	Optimum parameters	Stability	Activity Xylan	Activity CMC	Other parameters	References
*Fn*Cel5A	*Fervidobacterium nodosum*	*E. coli*	80–83 °C pH 5.0–5.5	Half-life of 48 h at 80 °C	No	440 U/mg	NM	[30]
*Tm*Cel5A	*Thermotoga maritima*	*E. coli*	90 °C pH 6.6	Stable at 85 °C	No	80 U/mg	*K*m = 0.24 mM with pnp-B-D-cellopentaoside	[31]
*Ct*Cel5E	*Clostridium thermocellum*	*E. coli*	50 °C pH 5.0	Not determined	Yes	736.2 ± 12.8 U	*K*m = 2.1 ± 0.2 (mg/mL); *kcat* = 1564.0 ± 69.1 min^−1^	[28]
Cel776*Ec*	Hot Spring Metagenome	*E. coli*	80 °C pH 5.0	Half-life of 3.55 h at 80 °C	Yes	2.12 U/mg	NM	This work
Cel776*Sc*	Hot Spring Metagenome	*S. cerevisiae*	80 °C pH 5.0	60% activity after 1 h at 70 °C	NM	NM	*K*m = 0.1716 µg µL^−1^; *V*max 0.6686 U µL^−1^	This work

NM: not measured

## Discussion

The role of thermozymes in lignocellulose degradation and their discovery process has been the subject of multiple reviews [2, 10, 12, 15, 32] as their biotechnological applications raises their interest as a promising environmentally friendly alternative to established processes along other advantages including operational cost reductions. Although endoglucanases [1, 3, 5, 6] and xylanases [8, 33–35] have multiple applications, where only one of the activities is required, synergism of both enzymes and even other accessory enzymes is also described for particular processes [9].

We identified an enzyme in this study as a putative endoglucanase belonging to the GH5 subfamily 25, named Cel776. Proteins in the GH5 family have a retaining mechanism of catalysis (the anomeric carbon configuration is conserved in the enzymatic reaction), with two Glu residues acting as the nucleophile and the electron donor (Glu144 and Glu260 in Cel776). Their structure consists in a (β/α)_8_-barrel, with some subfamilies featuring a modular domain architecture [36]. Modelling of the predicted protein Cel776 fitted the (β/α)_8_-barrel structure (Fig. 1) using the endoglucanase Cel5A from *F. nodosum* Rt17-B1 as template, which also belongs to subfamily 25 of GH5. Indeed, proteins in subfamily 25 include thermophilic and multifunctional enzymes [36] like said endoglucanase from *F. nodosum* Rt17-B1 which can also use β-d-glucan and galactomannan as substrates [37]; a endoglucanase/β-mannanase from *Thermotoga maritima* MSB8 [38], and a cellulase/xylanase from *Clostridium thermocellum* [28] (that appears as two entries in the database due to renaming of the species as *Hungateiclostridium thermocellum* [39] and *Acetibrivio thermocellus* [40]). Alignment to these characterized members of GH5 subfamily 25 with Cel776 confirmed conserved residues involved in the active site of the enzymes including the catalytic Glu pair (Fig. 2). Features such as a carbohydrate binding module present in some members of the subfamily such us the multifunctional endoglucanase from *C. thermocellum* [41] were not present in the Cel776, clearly evidenced in the Clustal Omega alignment, where nearly 300 amino acids at both ends of this protein did not align with the shorter GH5_25 endoglucanases analysed. Importantly, detection of a predicted N-glycosylation site on a key residue of the active site (His212) might be responsible for differences observed in the behaviour of the enzyme when expressed in the hosts *E. coli* and *S. cerevisiae* (Fig. 2). Moreover, our analysis also identified indels in the loop regions of the predicted model (Fig. 1C). In enzymes of the GH5 family, these loop regions are involved in substrate specificity in combination with the position of aromatic residues across the surface of the active site [42]. Knowledge on key residues that may be affecting thermostability, substrate specificity and catalytic rates open the possibility to improve Cel776 activity through strategies of protein engineering not unlike what have been achieved with other members of the family [6, 27–29]. Due to the extracellular expression of the enzyme Cel776*Sc* in the yeast host, the purification process of the enzyme was significatively simpler than the intracellular enzyme Cel776*Ec*. Activity assays confirmed that Cel776 is an endoglucanase, with multifunctional activity including xylanase activity with birchwood xylan as the substrate. The biochemical characterization also revealed that it is a thermozyme, with an optimal temperature of 80 °C that is maintained over a range of temperatures and capable of maintaining its activity for over 24 h incubation periods at 60 °C. Moreover, at its optimal temperature it showed a half-life of 3.55 h. The optimal pH was 5 and the enzyme activity and Cel776*Ec* was more severely affected by pH changes than Cel776*Sc*. There are many thermophilic endoglucanases that have been already characterized [3], and both the GH5 endoglucanase from *Acidothermus cellulolyticus* [43, 44] and the endoglucanase Cel5A from *T. maritima* [27] most closely resemble the temperature and pH optimums observed for Cel776. In addition, the behaviour of the enzyme towards variations on temperature and pH is similar to other GH5 family endoglucanases recently reported from metagenomic surveys [45, 46]. On the other hand, other enzymatic parameters are difficult to compare due to the heterogenous nature of the characterization of enzymatic activity (Table 1). Nevertheless, the enzymatic inhibition by zinc that Cel776 exhibited is present in many endoglucanases [45–47]. An effect of inhibition on enzymatic activity was observed for Cel766*Sc* but not for Cel776*Ec* when EDTA was added. Moreover, an enhancing effect of CaCl_2_ and MgCl_2_ additives on activity was observed for Cel776*Ec* and in Cel776*Sc* with MnCl_2_. Such an effect has been studied in several endoglucanases with calcium [48–50], where higher affinity for the substrate CMC is reported, whereas the effect of magnesium is reported in some enzymes, possibly because of a stabilization of the structure [47]. SDS remarkably inhibited the activity of both in Cel776*Ec* and Cel776*Sc*, an effect observed for other endoglucanases too [46]. The enzyme expressed in the yeast system was nonetheless more stable in this regard, which coupled with the extracellular expression that allows for a simpler purification process and the significatively larger yield obtained makes Cel776*Sc* a more desirable biocatalyst from a biotechnological perspective.

## Conclusions

In this study an endoglucanase from a hot spring metagenome was identified by sequence-based analysis and was successfully purified and biochemically characterized. The enzyme was multifunctional, mainly acting on CMC as an endoglucanase but also able to degrade xylan as a xylanase. Its high temperature optimum and thermotolerance are desirable traits regarding its potential as a biotechnological catalyst in processes such as biofuel production and saccharification of plant biomass. The enzyme expressed in yeast was more stable in presence of detergents, was simpler to purify and the yield was significatively higher, highlighting the advantages of this expression system.

## Methods

Metagenomic DNA was obtained from Muiño da Veiga hot spring, located in Galicia (northwest region of Spain), using the “Metagenomic DNA isolation kit for water” (Epicentre, USA) as described previously [51]. Up to 125L of hot spring water were processed for the extraction of high molecular weight DNA. NGS sequencing was performed using this metagenomic DNA. All NGS services were provided by Health in Code (A Coruña, Spain) using an Illumina Hi-Seq platform with a 2 × 100 pb sequence length.

### Quality filtering, assembly of short reads and decontamination

Sequence data was preprocessed with the prinseq-lite Perl script [52] to assess quality control: trim quality score threshold was 25 (right and left ends), minimum length was 60 bp, no unknown (N) basepair, sequence complexity by the entropy method (using a threshold of 70), and no sequences duplicates. Sequences corresponding to a pair-ended read were merged using the PEAR software [53] running on default settings. Quality-filtered merged reads in FASTA format and singletons (sequences that could not be merged from the sequencing pair) were all used as input for an assembly using a de-novo de Brujin graph based algorithm IDBA-UD [54] on default settings. The resulting contigs were decontaminated from sequences aligning to the human genome (contaminating sequences) using the Deconseq algorithm [55] with the NCBI GRCh38 data (RefSeq ID 884148) used as the reference database.

### Sequence upload to MG-RAST metagenomes database, identification of putative endoglucanase genes and bioinformatic analysis

The FASTA format file containing the contigs sequence data was uploaded to the MG-RAST [56] web service. The pipeline options for the upload were as follows: dereplication “yes”, screening “none”, length filtering “no”, ambiguous base filtering yes”, maximum ambiguous basepairs “5”.

An in-depth analysis was conducted to retrieve contig sequences containing genes coding for endoglucanases. The analysis tool from MG-RAST was used with the following parameters: e-value “5”, %-ident “60”, length “15”, min. abundance “1”, method “representative hit”. The database for gene product annotation employed was SEED Subsystems [57]. Annotated sequences at the function level were search with the term “Endoglucanase (EC 3.2.1.4)”. The resulting contigs containing putative genes coding for the selected activities were analyzed using ExPASy Translate Tool [58] to identify ORFs. The selected ORFs where then analyzed using NCBI BLASTp algorithm [59] on the default settings (protein–protein BLAST with BLOSUM62 matrix, cost of gap existence = 11 and of gap extension = 1) with the Non-redundant protein sequences database (nr). Selected putative proteins were structurally modelled using the SWISS-MODEL [60] web tool with the best matching template. Protein models were visualized with the PyMol software (Schrödinger LCC, USA) [61]. Protein parameters were predicted with the PROTPARAM algorithm from ExPASy [58]. Multiple sequence alignments used EMBL-EBI Clustal Omega [62] and sequences obtained from the UniProt Knowledgebase database [63]. Prediction of signal peptide features was performed using the SignalP 0.5 web service [64]. Prediction of glycosylation sites was achieved using the ScanProsite tool [65].

### Synthesis and cloning of a DNA fragment containing a putative endoglucanase gene in bacteria

The complete sequence of the gene with adapter sequences (provided as Additional file 1 in FASTA format) was ordered for synthesis (ThermoScientific, USA). The gene was provided as a DNA fragment readily available for cloning. The adapter sequences (*att*B sites) were added to both sequence ends to allow subcloning in the Gateway system (Invitrogen, USA). For subcloning the Gateway Technology with Clonase II (Invitrogen, USA) kit was used, following the manufacturer protocol. The *att*P sites containing vector pDNOR221 was used in the BP reaction as donor vector to generate an entry vector (*att*L site containing vector generated by the recombination of *att*B and *att*P sites). An equimolar amount of the endoglucanase gene fragment DNA and donor vector DNA was employed, and the reaction was carried out using TE Buffer pH 8 supplied in the kit and the Gateway BP Clonase II enzyme mix with an incubation at 25 °C for 1 h. The reaction was stopped adding Proteinase K solution to the reaction mix and incubating at 37 °C for 10 min. This reaction mix was used to transform chemically competent *E. coli* One Shot™ OmniMAX™ 2 T1^R^ (F*´* {*pro*AB *lac*I^q^ *lac*ZΔM15 *Tn*10(Tet^R^) Δ(*ccd*AB)} *mcr*A Δ(*mrr hsd*RMS*-mcr*BC) Φ 80(*lac*Z)ΔM15 Δ(*lac*ZYA-*arg*F)U169 *end*A1 *rec*A1 *sup*E44 *thi*-1 *gyr*A96 *rel*A1 *ton*A *pan*D) (Invitrogen, USA). A vial of chemically competent cells (50 µL) was thawed on ice and 1 µL of BP recombination reaction was added and mixed. The mixture was incubated for 30 min on ice, heat-shocked at 42 °C for 30 s and cooled on ice for 2 min. For recovery, cells were incubated with shaking at 37 °C for 1 h. Bacteria were plated on LB media (1% Bacto™ tryptone (BD, USA), 0.5% Bacto™ yeast extract (BD, USA), 0.5% NaCl, 1.5% Bacto™ Agar (BD, USA)) containing kanamycin for positive selection of transformant recombinant clones with an overnight period for growth. Correct recombination was tested by sequencing using the M13 sequencing primer pair, with the sequencing service provided by the Molecular Biology Unit from the Research Support Services of Universidade da Coruña (Spain). Plasmid DNA was recovered using the NZYMiniprep kit (NZYTECH, Portugal). The plasmid harbouring the insert (flanked by *att*L sites) was used for the next step of the protocol of LR recombination as supercoiled DNA. The destination vector was pDEST527 (which contains *att*R sites) and was also used as supercoiled DNA. The reaction was carried out in TE Buffer pH 8.0 with the Gateway LR Clonase II enzyme mix, with an incubation at 25 °C for 1 h, and it was stopped by adding 1 µL of Proteinase K and incubating for 10 min at 37 °C. Transformation was carried out using 1 µL of the LR reaction and a vial (50 µL) of chemically competent *E. coli* One Shot™ OmniMAX™ 2 T1^R^ following the same protocol for the thermal shock, recovery and plating as with the BP reaction. The LB media used for positive selection contained ampicillin instead of kanamycin. Correct insertion was verified plating on LB media containing both ampicillin and chloramphenicol, where negative selection occurred as chloramphenicol resistance is lost if a LR recombination takes place, and by sequencing both ends using the T7 sequencing primer pair. The resulting expression vector pDEST521 contained the insert with the gene for the putative endoglucanase flanked by *att*B sites and with a His(× 6) tag to facilitate purification and an inducible T7 promoter for controlled gene expression. DNA used for sequencing was also used to transform chemically competent *E. coli* T7 Express (New England Biolabs, USA) (*fhu*A2 *lac*Z::T7 *gene*1 [lon] *omp*T *gal sul*A11 R(*mcr*-73::*miniTn*10–*Tet*^S^)2 [dcm] R(*zgb*-210::*Tn*10–*Tet*^S^) *end*A1 Δ(*mcr*C-*mrr*)114::IS10). Transformation was carried out as described before changing the heat-shock step to 10 s at 42 °C and the incubation on ice to 5 min as recommended by the manufacturer. LB medium for positive selection contained ampicillin.

### Purification of Cel776Ec

Fresh cultures were stablished using an overnight inoculum at 37 °C from a single colony of *E. coli* T7 Express harbouring the pDEST527 vector and the cloned endoglucanase gene Cel776. The cells were harvested using a refrigerated centrifuge (4 °C) at maximum speed (8000 rpm) for 15 min and resuspended in resuspension buffer 100 mM Sodium Acetate pH 5, 100 mM NaCl and 1 mM DTT. Cell lysates were obtained using the sonicator with a setting of 70% amplitude, 3 min active time and pulses of 3 s ON and 7 s OFF. Cell debris was precipitated in a refrigerated (4 °C) centrifuge at maximum speed (8000 rpm) for 15 min, and the recovered supernatant was used as a crude enzyme extract. This extract was further purified using the following filtered and sonicator-degasified buffers: wash buffer 100 mM sodium acetate pH 5, 500 mM NaCl and 25 mM imidazol; elution buffer 100 mM sodium acetate pH 5, 100 mM NaCl and 300 mM imidazol. The extract was loaded in a niquel-sepharose resin-filled column HisTrap 5 mL (Cytiva, USA) and washed with 5 volumes (25 mL) of wash buffer in a single step (flow-through and wash fraction). A manual run with a peristaltic pump P-1 (Cytiva, USA) was conducted using a flow rate setting of 3.0 and pressure setting at 0.5. The elution buffer was diluted to obtain 10% elution buffer (30 mM imidazol) and 50% elution buffer (150 mM imidazol). Increasingly concentrations of elution buffer were loaded in the column to obtain the different elution fractions. Protein concentration determination using the Bradford method [66], enzymatic activity tests (described in the next section) and SDS–PAGE were all conducted on the fractions to monitor the purification process. A western-blot was also performed using anti-his-tag antibodies to detect the purified protein. The developed gel (14% acrylamide/bis-acrylamide) was cut and placed in contact with the western-blot membrane, and introduced in a sandwich such as fashion between two filter papers. For the bands transfer to the membrane, a cooled transfer buffer was employed with the following composition: Tris 25 mM pH 8.3, glycine 192 mM, 20% (v/v) methanol. The settings for the transfer were 300 mA for 60 min. After the transfer was completed, the membrane was incubated with a blocking solution consisting in Tris buffered saline (TBS), 0.1% (v/v) Tween 20 and 5% (w/v) BSA for 2 h in cold. After blocking, the membrane was incubated with a TBS solution containing 0.1% (v/v) Tween 20, 5% (w/v) BSA and HRP-conjugated 6XHis, His-Tag Monoclonal antibody (Proteintech, USA) in cold. To develop the chemiluminescence signal the components from Pierce ECL Plus Western Blotting Substrate kit (Fisher Thermo Scientific, USA) were employed, following the manufacturer protocol. Pictures were taken using a Chemidoc MP Imaging System (Bio-rad, USA), with a chemiluminescence setting for “high sensitivity” and exposition time “120 s” (several photos were taken in intervals), and a colorimetric photo was merged to include the protein ladder. The purified protein was concentrated to its final volume for activity assays using a Pierce Concentrator column with 10K Molecular Weight cutoff (Fisher Thermo Scientific, USA).

### Cloning of a DNA fragment in yeast and protein expression

PCR primers (Eurofins, Luxembourg) were designed for the gene fragment containing the Cel776 gene described in the cloning section for bacteria. This primer pair contained adapter sequences flanking the sequences to allow homologous recombination in yeast, the forward primer sequence was 5’AAAGAAGAAGGGGTACCTTTGGATAAAAgaatgctaacaagttgcagagaa3’ (homologous sequence in capital letters) and the reverse primer sequence was 5’tGGGACGCTCGACGGATCAGCGGCCGCTtactacttcccaagcgctgctgt3’. High fidelity Phusion DNA Polymerase (Thermo Scientific, USA) was used in the PCR reactions, annealing temperature was 70 °C. PCR clean-up was performed using the Gene JET Gel Extraction kit (Thermo Scientific, USA).

*Saccharomyces cerevisiae* BJ3505 [pep4: HIS3, prb-Δ1.6R HIS3, lys2-208, trp1-Δ101, ura 3–52, gal2, can1] (Eastman Kodak Company, USA) was transformed with plasmid YepFLAG-1 [*ampr ori 2μ FLAG TRP1*] (Eastman Kodak Company, USA), linearized with restriction enzymes *Xho*I and *Sal*I and the PCR amplified DNA fragment containing the Cel776 gene and adapter sequences for homologous recombination. A vial of yeast was incubated with the amplified PCR product and the linearized plasmid, following the commercial kit protocol Frozen-EZ Yeast Transformation II (Zymo Research, USA). Transformed yeast were incubated at 30 °C in CM without tryptophan [67] plates for 2 to 3 days. Up to 15 colonies were PCR tested for correct cloning of the desired DNA fragment containing Cel776.

For protein expression, the selected clone containing the Cel776*Sc* gene was cultured in culture flasks containing YPHSM (1% yeast extract, 8% Bacto™ peptone (BD, USA), 1% glucose, 3% glycerol) medium, at 30 °C for 4 days. As the protein was expressed in the extracellular medium, yeast cultures were centrifuged for 5 min at 8000 rpm and the supernatant was kept at 4 °C. This supernatant (containing the Cel776*Sc* enzyme) was concentrated with Amicon ultrafiltration devices (Merck Millipore, Germany) with a cutoff of 3KDa, with centrifugation at 8000 rpm until the supernatant was sufficiently concentrated for biochemical assays.

### Biochemical characterization of a novel endoglucanase

Endoglucanase activity for both Cel776*Ec* and Cel776*Sc* was tested using the reducing sugars method with dinitrosalicylic acid (DNS) with soluble carboxymethylcellulose (CMC, Sigma-Aldrich, USA) as the substrate [68]. 5 µL of the purified enzyme were mixed with 45 µL of reaction buffer and 50 µL of 1% CMC. Reaction time was 30 min, and the reaction was stopped by adding 150 µL DNS and incubating for 5 min at 100 °C. Then, 1 mL ice-cold milliQ water were added and the tubes were placed on ice until the absorbance was read at wavelength 540 nm in 96 well-plates (Sinergy H1 Hybrid Multi-Mode Reader, BioTek, USA). All conditions were assayed in triplicates and blanks were also assayed, where the volume of purified enzyme was replaced with the same volume of milliQ water. An enzymatic unit is defined as the amount of enzyme that releases a micromol of glucose per minute in the reaction conditions. A glucose standard was prepared to perform a linear regression of the relationship between absorbance and glucose concentration.

The optimal temperature was evaluated at pH 5 (reaction buffer was 100 mM sodium acetate pH 5 at 60 °C) in the range between 30 and 90 °C with a 10 °C interval. The optimal pH was evaluated at 60 °C in a range between 4 and 9 with an interval of 1. Reaction buffers for the pH intervals were 100 mM Sodium Acetate buffer for the range 4–6, 100 mM Sodium Phosphate buffer for the range 6–8 and 100 mM Tris HCl buffer for the range 8–10. Thermostability was assayed at optimal pH and temperature after incubation of the enzyme at 60 °C, 70 °C, 80 °C and 90 °C for a set amount of time. Alternative substrates were assayed at 1% concentration (w/v), insoluble microcrystalline cellulose AVICEL (Sigma-Aldrich, USA), beechwood xylan (Megazyme, Ireland), filter paper (Scharlab, Spain), cotton (Corman Spa, Italy) and starch (Sigma-Aldrich, USA), with CMC as control substrate. The following additives and detergents were tested to evaluate the effect on the enzymatic activity (final concentrations 5 mM): CaCl_2_, ZnSO_4_, MgCl_2_, MnCl_2_, EDTA and SDS. For Cel776*Sc* an enzyme kinetics determination was performed using various concentrations of the CMC substrate (0.5; 0.75; 1; 1.25; 1.5; 2 and 5 mM).

## Supplementary Information

**Additional file 1.** Complete sequence of the gene encoding Cel776 with adapter sequences for subcloning in the Gateway system.

**Additional file 2: Figure S1.** Structural alignment of the model generated with Swiss Model using as template the endoglucanase from *Fervidobacterium nodosum* (FnCel5A) in complex with substrate alpha-D-glucopyranose (3rjy.1.A, green); the endoglucanase from *Thermotoga maritima* (TmCel5A) in complex with cellobiose (3azr.1.A, yellow) and the inactive mutant endoglucanase from *Clostridium thermocellum* [4u5i.1.A, CtCel5E (E314A)] in complex with xylobiose (light blue). Substrates and conserved residues linked to the catalytic activity are represented in ball-and-stick model.

## Data Availability

The data sets generated and analysed during the current study are available in the MG-RAST repository, https://www.mg-rast.org/linkin.cgi?project=mgp84238.
