# Supplementary material for: Characterization of a novel thermophilic metagenomic GH5 endoglucanase heterologously expressed in Escherichia coli and Saccharomyces cerevisiae
Source: Biotechnol Biofuels Bioprod. 2022 Jul 7;15:76. doi: 10.1186/s13068-022-02172-4 (PMC9264688; doi:10.1186/s13068-022-02172-4)
Supplement: Supplementary file 2 — Additional file 2: Figure S1. Structural alignment of the model generated with Swiss Model using as template the endoglucanase from Fervidobacterium nodosum (FnCel5A) in complex with substrate alpha-D-glucopyranose (3rjy.1.A, green); the endoglucanase from Thermotoga maritima (TmCel5A) in complex with cellobiose (3azr.1.A, yellow) and the inactive mutant endoglucanase from Clostridium thermocellum [4u5i.1.A, CtCel5E (E314A)] in complex with xylobiose (light blue). Substrates and conserved residues linked to the catalytic activity are represented in ball-and-stick model. [file 13068_2022_2172_MOESM2_ESM.docx]

**
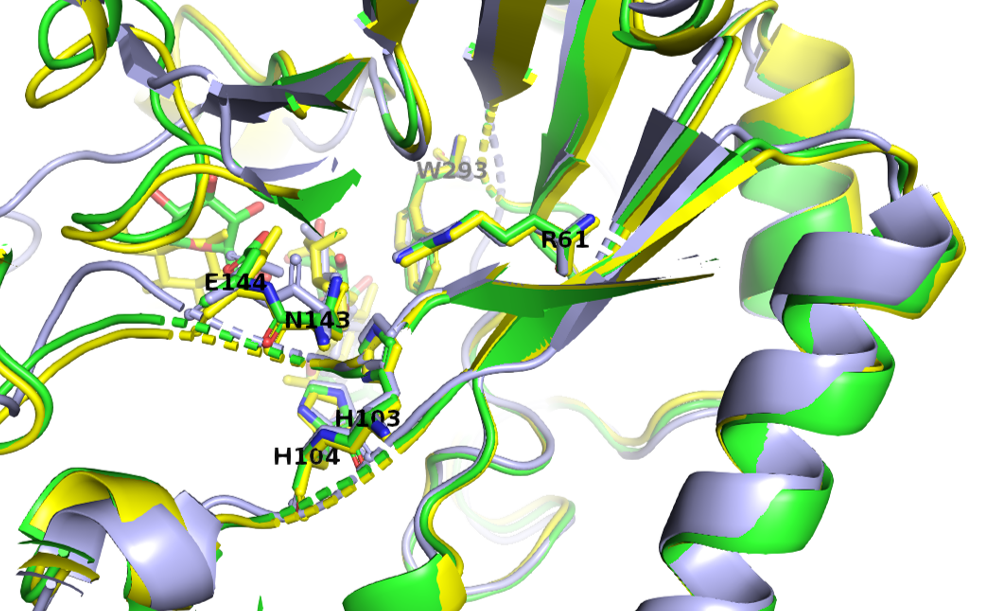
**

**Figure S1.** Structural alignment of the model generated with Swiss Model using as template the endoglucanase from *Fervidobacterium nodosum* (FnCel5A) in complex with substrate alpha-D-glucopyranose (3rjy.1.A, green); the endoglucanase from *Thermotoga maritima* (TmCel5A) in complex with cellobiose (3azr.1.A, yellow) and the inactive mutant endoglucanase from *Clostridium thermocellum* [4u5i.1.A, CtCel5E (E314A)] in complex with xylobiose (light blue). Substrates and conserved residues linked to the catalytic activity are represented in ball-and-stick model.
